# Highly Pathogenic Avian Influenza A(H5N1) Virus Infection among Workers at Live Bird Markets, Bangladesh, 2009–2010

**DOI:** 10.3201/eid2104.141281

**Published:** 2015-04

**Authors:** Sharifa Nasreen, Salah Uddin Khan, Stephen P. Luby, Emily S. Gurley, Jaynal Abedin, Rashid Uz Zaman, Badrul Munir Sohel, Mustafizur Rahman, Kathy Hancock, Min Z. Levine, Vic Veguilla, David Wang, Crystal Holiday, Eric Gillis, Katharine Sturm-Ramirez, Joseph S. Bresee, Mahmudur Rahman, Timothy M. Uyeki, Jacqueline M. Katz, Eduardo Azziz-Baumgartner

**Affiliations:** icddr,b, Dhaka, Bangladesh (S. Nasreen, S.U. Khan, S.P. Luby, E.S. Gurley, J. Abedin, R.U. Zaman, B.M. Sohel, M. Rahman, K. Sturm-Ramirez, E. Azziz-Baumgartner);; Centers for Disease Control and Prevention, Atlanta, Georgia, USA (S.P. Luby, K. Hancock, M.Z. Levine, V. Veguilla, D. Wang, C. Holiday, E. Gillis, K. Sturm-Ramirez, J.S. Bresee, T.M. Uyeki, J.M. Katz, E. Azziz-Baumgartner);; Institute of Epidemiology, Disease Control and Research, Dhaka (M. Rahman)

**Keywords:** seroprevalence, seroconversion, incidence, influenza, subtype H5N1, viruses, live bird market, poultry worker, Bangladesh, transmission, highly pathogenic avian influenza, risk factors

## Abstract

The risk for influenza A(H5N1) virus infection is unclear among poultry workers in countries where the virus is endemic. To assess H5N1 seroprevalence and seroconversion among workers at live bird markets (LBMs) in Bangladesh, we followed a cohort of workers from 12 LBMs with existing avian influenza surveillance. Serum samples from workers were tested for H5N1 antibodies at the end of the study or when LBM samples first had H5N1 virus–positive test results. Of 404 workers, 9 (2%) were seropositive at baseline. Of 284 workers who completed the study and were seronegative at baseline, 6 (2%) seroconverted (7 cases/100 poultry worker–years). Workers who frequently fed poultry, cleaned feces from pens, cleaned food/water containers, and did not wash hands after touching sick poultry had a 7.6 times higher risk for infection compared with workers who infrequently performed these behaviors. Despite frequent exposure to H5N1 virus, LBM workers showed evidence of only sporadic infection.

## Abstract 2

Evidence of infection was low despite frequent exposure to infected poultry and low use of personal protective equipment.

Human infections and deaths caused by highly pathogenic avian influenza A (H5N1) viruses in several countries (*1*); by A(H9N2) virus in Bangladesh (*2*); and by A(H7N2), A(H7N9), A(H9N2), and A(H10N8) viruses in China (*3**–**5*) reflect the persistent public health threat posed by different avian influenza A virus subtypes. Subtype H5N1 virus remains endemic among poultry in Bangladesh, China, Egypt, Indonesia, and Vietnam (*6*). Among these countries the first human cases of H5N1 virus were identified in China and Vietnam during 2003 (*1*). The seroprevalence of antibodies against H5N1 virus among poultry workers was 0%–4% in Bangladesh, China, Indonesia, and Vietnam during 2001–2009 (*7**–**13*); published data on seroprevalence among poultry workers in Egypt are not available. Beyond the countries where H5N1 is endemic, 0%–10% seroprevalence has been reported among poultry workers in Nigeria; South Korea; Thailand; and Hong Kong, China (*14**–**17*). The incidence of H5N1 virus infection among occupationally exposed populations has not been determined in countries where the virus is endemic or nonendemic.

In Bangladesh, a country with a population density of 964/km^2^ and 257 million poultry (*18**,**19*), H5N1 virus infection was first detected among poultry in 2007. By the end of 2013, the country had reported 549 outbreaks among poultry to the World Organisation for Animal Health (*20*). The first human case of H5N1 virus infection in Bangladesh was identified during 2008 (*21*). Live bird markets (LBMs) are often associated with poultry-to-human transmission of H5N1 virus (*22*). For example, butchering and exposure to sick poultry were associated with detection of H5 antibody among LBM workers in Hong Kong (*17*). In one study, workers from 16 LBMs in Bangladesh were rarely observed using personal protective equipment (PPE) or washing their hands during the handling of poultry, suggesting a high likelihood of exposures to H5N1 virus (*23*). Data are limited on the risk for avian influenza A virus infections among poultry workers in Bangladesh (*7*).

Seroprevalence studies among humans yield information about how many persons have serologic evidence of infection at a certain point and time, but they do not provide information about when people became infected or the risk for infection with prolonged exposures to contaminated animals or environments. Studies designed to estimate the rate of seroconversion of antibodies to H5N1 virus among poultry workers may also help elucidate the risks of poultry-to-human transmission of H5N1 virus in countries, such as Bangladesh, where H5N1 virus is endemic among poultry. Such information may help public health officials develop, prioritize, and reinforce prevention and control strategies. During 2009–2010, a total of 61 H5N1 outbreaks, resulting in the culling of 220,432 birds, were reported among poultry in Bangladesh (*24*); no human cases were identified during this period. We followed a cohort of LBM workers in Bangladesh to determine the seroprevalence of antibodies to H5N1 virus, the incidence of seroconversion, and risk factors for poultry-to-human transmission of H5N1 virus.

## Methods

### Study Sites

We conducted this study among workers in 12 LBMs in 4 districts of Bangladesh: 8 in Dhaka, 2 in Chittagong, and 1 each in Netrokona and Rajshahi. We selected these LBMs because they served as sentinel sites for existing avian influenza surveillance throughout the study period; surveillance included the monthly collection of poultry and environmental samples (*25**,**26*). The samples were tested for influenza A and subtype H5 by using real-time reverse transcription PCR (*27*). By April 2009, H5N1 virus was detected from farms in 47 of 64 districts in Bangladesh, including the 4 districts where the LBMs in our study were located (*20*).

The LBMs in Dhaka, which were open daily from 6:00 AM to midnight, sold chickens, ducks, geese, and quail. The workers slaughtered, defeathered, eviscerated, and sold the poultry. LBMs outside Dhaka were in rural subdistricts and were open once or twice a week. Backyard poultry farmers and, occasionally, commercial poultry farmers sold poultry at these LBMs.

### Poultry Worker Enrollment and Baseline Data Collection

We aimed to recruit ≈400 workers. All workers 18–59 years of age were eligible for enrollment. This age limit maximized the specificity of detection of H5N1 virus antibodies by microneutralization assay with confirmatory Western blot because the specificity of these assays is lower among older adults (*28*). The field team prepared a list of 721 eligible poultry workers present at the LBMs from 8:00 AM to 5:00 PM.

In 2009, we enrolled a convenience sample of consenting workers from rural subdistrict LBMs during May–June and from urban Dhaka LBMs during October–November, when poultry surveillance became operational (Figure). The poultry workers were enrolled as a closed cohort. The field team used a structured questionnaire (Technical Appendix 1 Figure 1) to collect demographic data and information about any history of chronic medical conditions; habits involving frequent hand-to-mouth contact (i.e., smoking, smokeless tobacco use, and betel leaf/nut use); the location of poultry handling; and practices that may have placed the workers at risk for H5N1 virus infection (i.e., not wearing PPE, eating while working with poultry, holding or carrying poultry, and eating raw or undercooked poultry or eggs). Medical technologists collected a 10-mL blood specimen from each study participant.

**Figure:**
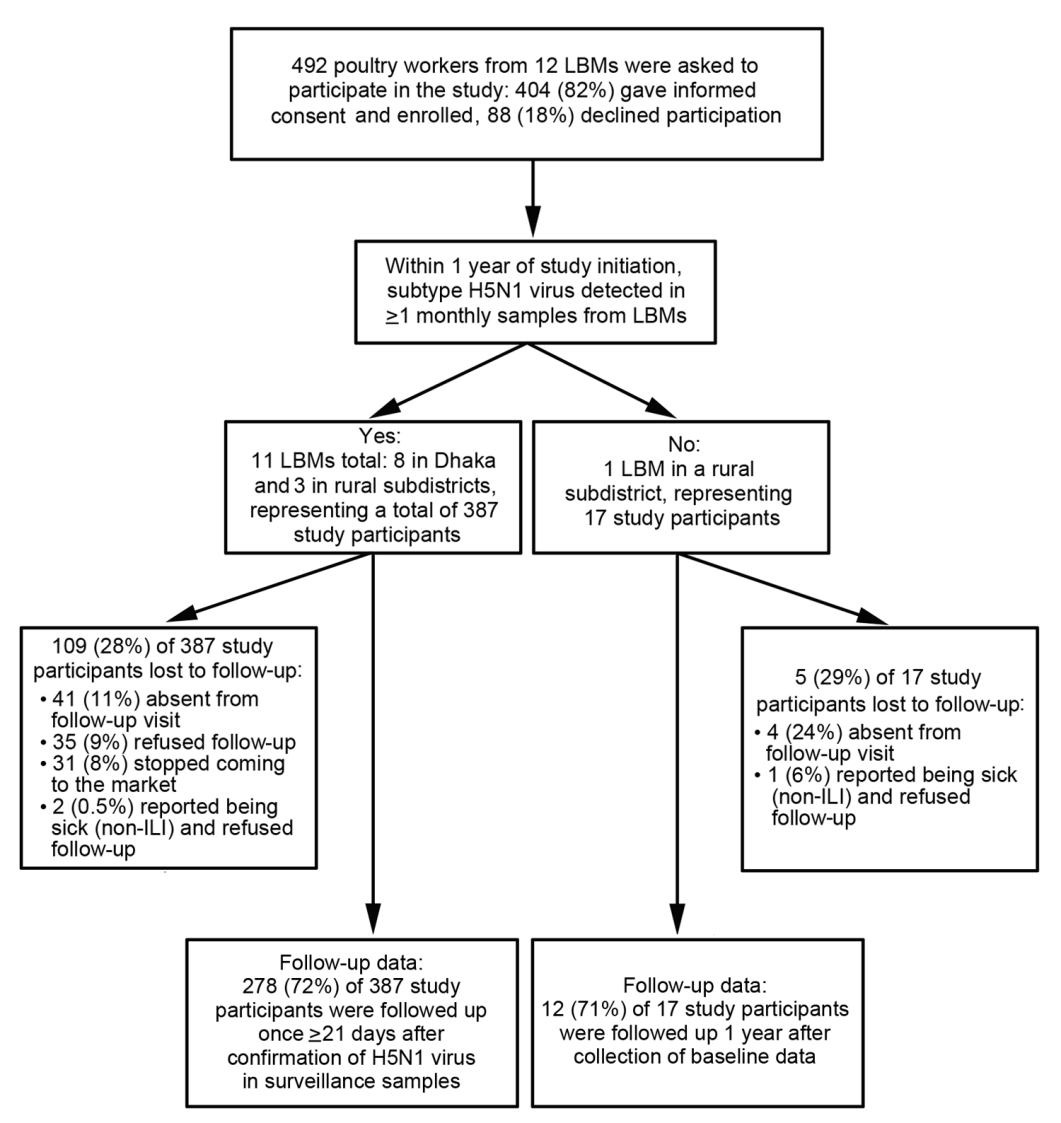
Enrollment and data for participants in a study of influenza A(H5N1) virus infection among workers at live bird markets (LBMs), Bangladesh, 2009–2010. ILI, influenza-like illness.

### Follow-up Data Collection

During January–April 2010, which included the peak period of H5N1 virus circulation among poultry (*26*), we followed up with study participants one time. Follow-up occurred >21 days after virus was first detected through poultry surveillance (*25*) or 1 year after enrollment if H5N1 virus was not detected in an LBM where a study participant worked (Figure). At follow-up, the field team collected information about any history of influenza-like illness (i.e., subjective or measured fever and cough or sore throat) and shortness of breath or difficulty breathing within the 21 days before the follow-up visit and about exposure to sick poultry and precautions taken in the 3 days before respiratory symptom onset (if applicable) or 7 days before collection of the H5N1 virus–positive poultry or environmental surveillance sample (Technical Appendix 1 Figure 2). In LBMs where H5N1 virus was not detected through poultry surveillance within 1 year after baseline data collection, the field team obtained follow-up data during June 2010, using a questionnaire similar to the one used at baseline. Medical technologists collected a 10-mL blood specimen from all participants during follow-up.

### Data Collection from Nonpoultry Workers

In 2010, to get a sense of the baseline seroprevalence rate in a seemingly lower-risk population and to optimize the interpretation of the microneutralization assay results, we obtained samples from a group of nonpoultry workers. We enrolled a convenience sample of nonpoultry workers (18–59 years of age) from 3 accommodating nongovernmental organizations; these persons worked in Dhaka, did not own poultry, and had not participated in studies associated with influenza or other animals since the first detection of H5N1 virus among poultry in Bangladesh during 2007. During July and August 2010, using a structured questionnaire (Technical Appendix 1 Figure 3), the field team collected demographic data and information about any history of chronic medical conditions; habits involving frequent hand-to-mouth contact (e.g., smoking, smokeless tobacco use, and betel leaf/nut use); and lifetime history of ever handling poultry. Medical technologists collected a 10-mL blood specimen from each nonpoultry worker.

### Processing of Blood Specimens and Laboratory Analysis

All blood specimens were transported to the icddr,b laboratory in Dhaka on frozen cold packs at 2°C –8°C. Specimens collected outside Dhaka were centrifuged at the end of each day to separate serum and then transported. Specimens collected in Dhaka were transported to and centrifuged at icddr,b the same day. All serum samples were split into 3 aliquots and stored at icddr,b at −70°C. One aliquot was shipped on dry ice to the Influenza Division at the Centers for Disease Control and Prevention (CDC; Atlanta, GA, USA) for H5N1 serologic testing.

We performed the microneutralization assay as previously described (*28**,**29*), using H5N1 clade 2.2 (A/Bangladesh/3233/2011) virus, the most common strain identified through surveillance in Bangladesh during the study period. Serial 2-fold dilutions of serum (1:10–1:1,280) were tested. Samples that tested positive by microneutralization assay were also tested by a confirmatory Western blot assay against influenza strain recombinant hemagglutinin A/bar-headed goose/Qinghai/1A/2005 (clade 2.2). Samples with positive assay results or that demonstrated evidence of seroconversion against H5N1 virus were also tested by microneutralization and hemagglutination inhibition assays against pandemic influenza A(H1N1)pdm09 virus strain A/Mexico/4108/2009 (H1N1) to exclude potential serum antibody cross-reactivity. Serum samples that had high titers to A(H1N1)pdm09 virus were adsorbed with A(H1N1)pdm09 virus and then retested by microneutralization for reactivity to H5N1 virus. A seropositive result was defined as an H5N1 virus microneutralization titer >40 (equivalent to World Health Organization criteria >80) and confirmation by an H5-specific Western blot (*28**–**30*). Seroconversion against H5N1 virus was defined as detection of a >4-fold rise in microneutralization antibody titer between the initial serum sample and a paired second serum sample, with the second sample achieving a titer >40. Serum samples were tested >2 times by using the microneutralization assay. Microneutralization titers were expressed as the geometric mean of replicate titers.

### Estimating Seroprevalence and Seroconversion

We calculated the proportion of poultry workers and nonpoultry workers that were seropositive at baseline, the proportion of poultry workers that seroconverted against H5N1 virus, and 95% CIs of the proportions, assuming binomial distribution. We calculated the incidence of seroconversion against H5N1 virus among workers with paired serum samples who were from LBMs where H5N1 virus was detected through poultry surveillance; workers who were seropositive at baseline were excluded. We calculated the incidence by dividing the number of seroconversions by the person-time each participant contributed to the study between baseline and follow-up data collection and calculated 95% CIs, assuming a Poisson distribution. To be conservative, we assumed that workers were at risk of acquiring H5N1 virus between baseline and follow-up serum collection even though the LBM may have been free of H5N1 virus during some of that period. We extrapolated our calculated incidence of seroconversion among the participating poultry workers to estimate the annual number of poultry workers infected with H5N1 virus among the 721 eligible workers. To compare characteristics between poultry workers and nonpoultry workers, exposure to poultry, and use of PPE between workers who were followed versus those who were lost to follow-up, we performed the 2-sample Wilcoxon rank-sum test and 2-sample test of proportions.

### Statistical Analysis of Potential Risk Factors for H5N1 Virus Infection

We assessed risk factors for H5N1 virus infection (seropositivity or seroconversion) only among poultry workers with paired serum samples. Candidate risk factors were collinear, precluding the use of a regression model. Therefore, we performed the Kaiser-Meyer-Olkin test to assess the applicability of factor analysis for this dataset (*31*) and selected sets of common behaviors that explained >90% of variance among the candidate variables. Using the contribution of individual behavior (factor loading) as the basis, we grouped the behaviors into 3 sets and estimated the factor score for each set. Poultry workers with scores above median and those with scores below median were classified, respectively, as frequently and infrequently engaging in these sets of behaviors. We used a log-linear model, adjusted for clustering at the market level, to calculate risk ratio of serologic evidence of H5N1 virus infection for each set of behaviors between workers who were seropositive or seroconverted and those who were not seropositive and did not seroconvert against H5N1 virus (*32*). We applied robust sandwich SE estimation strategy to account for the correlation (*33*).

### Protection of Human Subjects

We obtained written informed consent from all participants before enrollment. Institutional review boards at icddr,b and CDC approved the study protocol.

## Results

We enrolled 404 LBM poultry workers in the study: 332 from Dhaka and 72 from rural subdistricts. The percentage of refusals was 18% (71/403) in LBMs in Dhaka and 17% (15/89) in those outside Dhaka. Most refusals were due to an unwillingness to provide a serum sample. We collected data from 101 nonpoultry workers, all of whom were from Dhaka. Overall, compared with nonpoultry workers, poultry workers were younger (median age 28.0 years [interquartile range (IQR) 22.5–38.0 y] vs. 36.0 years [IQR 32–40 y]) and more likely to be male (100% vs. 78%) and to smoke (58% vs. 34%) (p<0.001) (Table 1).

**Table 1:** Characteristics of live bird market workers and nonpoultry workers, Bangladesh, 2009–2010*

Characteristic	Poultry workers, n = 404	Nonpoultry workers, n = 101	p value
Male sex	404 (100)	79 (78)	<0.001†
Median age, y (IQR)	28 (22–38)	36 (32–40)	<0.001‡
Smoke tobacco	236 (58)	34 (34)	<0.001†
Median duration of smoking, y (IQR)	8 (4–16)	15 (9–20)	0.003‡
Use betel leaf or nut	151 (37)	22 (22)	0.003†
Use smokeless tobacco	15 (4)	1 (1)	0.2
Have chronic medical condition§	28 (7)	11 (11)	0.2

*Data are no. (%) persons except as indicated. IQR, interquartile range. †Value for 2-sample test of proportion. ‡Value for 2-sample Wilcoxon rank-sum test. §Conditions such as asthma; diabetes; chronic heart, lung, kidney, and liver disease; immune disorders; and cancer.

### H5N1 Serologic Testing Results

Of 404 poultry workers, 9 (2%) were seropositive for H5N1 virus antibodies at baseline (95% CI 1%–4%). During November 2009–March 2010, routine icddr,b poultry surveillance identified H5N1 virus at 11 (92%) of the 12 LBMs and in 25 (93%) of 27 monthly samples. We obtained a second blood specimen from 278 (72%) of 387 participating poultry workers from the 11 LBMs (Technical Appendix 2 Table 1). Because of a delay in the availability of laboratory results for poultry and environmental surveillance samples, the median interval between detection of H5N1 virus at LBMs and collection of a second blood sample from poultry workers at the corresponding LBM was 56 days (IQR 49–61 days).

Of 9 seropositive poultry workers at baseline, 5 remained seropositive and 1 was seronegative for H5N1 virus antibodies at follow-up (Technical Appendix 2 Figure); the remaining 3 workers were lost to follow-up. Six (2%) of 284 poultry workers seroconverted during the study period (95% CI 1%–5%) (Table 2). Six other workers met the criteria for seropositivity in the follow-up serum samples, but they were not considered to have seroconverted because baseline titers were >10 and a >4-fold rise in titer was not achieved.

**Table 2:** Characteristics of live bird market workers with evidence of seroconversion against avian influenza A(H5N1) virus, Bangladesh, 2009–2010*

Characteristic	PW1	PW2	PW3	PW4	PW5	PW6
Personal characteristic						
Age, y	28	20	22	24	38	19
Smoke tobacco	+	−	+	−	−	+
Have chronic medical condition	−	−	−	−	−	−
Exposure to poultry or virus						
Handle sick poultry	+	+	+	+	+	+
Perform tasks with sick poultry						
Transport poultry	+	+	+	+	−	+
Feed poultry	−	+	+	+	−	+
Clean feeding tray	−	−	+	+	−	+
Clean water container	−	+	+	+	−	+
Medicate sick poultry	−	−	+	−	−	−
Separate sick poultry	−	+	+	+	+	+
Slaughter poultry	−	+	+	+	−	+
Defeather poultry	−	+	−	+	−	+
Eviscerate poultry	−	+	−	+	−	+
Stuff poultry into bags	+	−	−	+	+	+
Clean feces from pen	−	−	+	−	−	+
Hand-carry sick poultry or held poultry on lap	+	+	+	+	+	+
Carry baskets containing sick poultry on head	−	−	−	−	−	−
Eat raw/undercooked poultry or eggs	−	+	+	−	−	−
Precautions taken when handling sick poultry						
Use personal protective equipment†	−	−	−	−	−	−
Wash hands at the market after working with the poultry	+	+	−	+	−	−
Change clothes upon returning home	+	+	+	+	+	+
Presence of influenza-like illness in past 21 d	−	−	−	−	−	−
Interval, d, between detection of virus–positive surveillance sample and follow-up collection of serum sample from worker	49	50	54	56	91	26
Neutralizing antibody titer, geometric mean‡						
Baseline	5	10	5	5	5	5
Follow-up	40	61	49	40	66	67

*PW, poultry worker; +, characteristic present; −, characteristic not present. †Apron, gloves, dedicated coveralls, cloth mask, and boots. ‡By microneutralization assay, using influenza A/Bangladesh/3233/2011 (H5N1, clade 2.2) virus.

H5N1 virus was not detected by routine poultry surveillance in 1 subdistrict LBM during the study period. We collected follow-up data from 12 (71%) of 17 participating poultry workers at this LBM 1 year after baseline enrollment, and all 12 were seronegative for H5N1 virus at enrollment and follow-up. The overall seroprevalence of antibodies to H5N1 virus among poultry workers from all LBMs during the study period was 5% (20/404, 95% CI 3%–7%). In comparison, none of the 101 nonpoultry workers was seropositive (95% CI 0%–4%).

### Incidence of Seroconversion

In LBMs where H5N1 virus was detected through routine poultry surveillance, we followed 278 poultry workers, of whom 266 were H5N1 virus–seronegative at baseline. These 266 workers contributed 30,043 days (≈82 years) of observation between the collection of paired blood samples, resulting in an incidence of 7 cases/100 poultry worker–years (95% CI 3–16). Using this incidence, we estimate that the annual incidence of H5N1 virus infection after exposure to H5N1 virus at the study LBMs was 50 cases per 721 enlisted poultry workers.

### Risk Factors for H5N1 Virus Infection

Seventeen (94%) of the 18 workers who were seropositive or seroconverted against H5N1 virus and 180 (66%) of the 272 seronegative workers reported exposure to poultry through >1 activity. None of the workers who were seropositive or who seroconverted reported exposure to poultry at home, at their farm, or at another place.

Three sets of behaviors explained 95% of the variability among risk behaviors at baseline and follow-up. However, the risk for H5N1 virus infection (risk ratio) was not equal for each set of behaviors (Technical Appendix 2 Table 2). The set of behaviors with the highest risk ratio consisted of feeding poultry, cleaning feeding trays and water containers, not washing hands after working with sick poultry, and cleaning feces from pens; this set of behaviors was classified as high exposure. The set of behaviors with the second highest risk ratio consisted of slaughtering, defeathering, eviscerating, collecting or transporting feces, and stuffing poultry into bags; this set of behaviors was classified as medium exposure. The set of behaviors with the lowest risk ratio included smoking, medicating poultry, isolating sick poultry, and eating raw or undercooked poultry or eggs; this set of behaviors was classified as low exposure.

Poultry workers who frequently performed high-exposure behaviors had a 7.6 times higher risk for H5N1 virus infection compared with poultry workers who infrequently performed high-exposure behaviors when they also infrequently performed medium-exposure behaviors (p<0.001) (Table 3). Poultry workers who frequently performed medium-exposure behaviors had a 5.1 times higher risk of H5N1 virus infection compared with poultry workers who infrequently performed medium-exposure behaviors when they also infrequently performed high-exposure behaviors (p = 0.002).

**Table 3:** Risks for testing seropositive or seroconverting against avian influenza A(H5N1) virus among live bird market workers, Bangladesh 2009–2010*

Characteristic/behavior	Poultry workers			Regression model		
	Seronegative, n = 272	Seropositive or seroconverted, n = 18		Simple RR (95% CI)	Multiple RR (95% CI)	p value†
Median age, y (IQR)	27 (23–38)	27 (20–30)		0.9 (0.9–1.0)	0.9 (0.9–1.1)	0.8
Risk behavior						
High exposure				4.8 (0.8–28.2)	7.6 (2.8–20.9)	<0.001
Feed poultry	196 (72)	17 (94)				
Clean feeding tray	156 (57)	15 (83)				
Clean water container	155 (57)	16 (89)				
Clean feces from poultry pen	125 (46)	14 (78)				
Do not wash hands after handling sick poultry	133 (49)	10 (56)				
Medium exposure				3.5 (0.8–14.7)	5.1 (1.8–14.1)	0.002
Slaughter poultry	198 (73)	17 (94)				
Defeather poultry	142 (52)	15 (83)				
Eviscerate poultry	143 (53)	15 (83)				
Collect or transport poultry feces	53 (19)	1 (6)				
Stuff poultry into bags	113 (42)	14 (78)				
Low exposure				1.0 (0.3–3.3)	–	–
Smoke	159 (58)	7 (39)				
Medicate poultry	15 (6)	2 (11)				
Isolate sick poultry	130 (48)	10 (56)				
Eat raw/undercooked poultry or eggs	103 (38)	6 (33)				
Risk of infection from						
Medium-exposure behaviors when frequently performing both medium- and high-exposure behaviors‡	–	–		–	1.4 (0.3–6.2)	0.6
High-exposure behaviors when frequently performing both high- and medium-exposure behaviors‡	–	–		–	2.1 (0.4–12.9)	0.4

*Data are no. (%) except as indicated. IQR, interquartile range; RR, risk ratio; –, not applicable. †Value for multivariate model. ‡The ratio of RR for interaction between medium- and high-exposure behaviors was 0.3 (1.4/5.1 for medium-exposure behaviors and 2.1/7.6 for high-exposure behaviors (95% CI 0.08–0.88; p = 0.031).

## Discussion

Our study demonstrates that, despite frequent exposure to infected poultry and low PPE use, LBM workers in Bangladesh had low serologic evidence of H5N1 virus infection. These results also suggest that cross-sectional seroprevalence studies may underestimate the risk for H5N1 virus infection if conducted outside the peak time for H5N1 virus circulation or if samples are obtained from infected workers long after exposure to the virus (i.e., when antibody titers have declined below the seropositive threshold).

Two percent of poultry workers were H5N1 virus–seropositive at baseline. This finding suggests that previous infection with H5N1 virus was uncommon despite the frequent exposure of workers to poultry. One of the workers who was seropositive at baseline became seronegative at follow-up, possibly because neutralizing antibodies decreased below the threshold for laboratory detection (*34*). The overall 5% seroprevalence of H5N1 virus antibody among poultry workers in our study is similar to the 4% seroprevalence among LBM workers in Vietnam in 2001 (*13*) but higher than the <1% seroprevalence reported among LBM workers from Bangladesh, Nigeria, Indonesia, and China during 2005–2009 (*7**,**9**,**11**,**14*). This finding suggests that human infection with H5N1 virus among heavily exposed workers at LBMs occurs infrequently but may be more common than previously reported. Routine poultry surveillance that included subdistrict LBMs in our study detected H5N1 virus from a higher proportion of poultry and environmental samples collected in 2011 than in 2009 and 2010 (3.8% vs. 0.4% and 0.5%, respectively) (*26*). Indeed, we would expect an increase in seroprevalence of H5N1 virus antibodies or seroconversion rates among exposed poultry workers during periods with increased H5N1 virus activity among poultry (*35*). Nevertheless, it is unclear whether the current proportion represents a substantive opportunity for virus reassortment and the generation of a novel virus with pandemic potential.

We identified 2 sets of correlated behaviors that increased the risk of acquiring H5N1 virus infection among poultry workers. Frequently performing high-exposure behaviors was associated with 1.5 times higher risk of acquiring H5N1 virus infection compared with performing medium-exposure behaviors. Only butchering and exposure to ill poultry were associated with H5 seropositivity among LBM workers performing >1 poultry-related task in Hong Kong (*17*). The single seropositive LBM worker in China also reported slaughtering birds for 5 years (*36*). The use of PPE while performing high-exposure behaviors and frequent handwashing may reduce the risk for H5N1 virus infection (*37*). Nevertheless, because poultry workers handle poultry throughout the workday, it may be challenging for them to use PPE every time they have contact with poultry or their feces (*38*). Virus exposure and subsequent infection via mucous membranes and the respiratory tract may also be reduced among workers if they avoid touching their eyes, mouth, and nose while at work. Formative research would be helpful to explore if and how environmental controls (e.g., handwashing stands, improved ventilation flow, scalding pots); improved poultry handling techniques (e.g., slaughtering poultry inside plastic bags); and improved PPE (e.g., more accessible, cost-effective, and better tolerated equipment) could help decrease the risk for virus transmission at LBMs.

In Bangladesh, most identified cases of H5N1 virus infection in humans have been asymptomatic or mildly symptomatic (*2**,**21*). However, in 2013, the potential for severe and fatal illness from H5N1 virus infection in Bangladesh was highlighted by a fatal case in a child who had been exposed to infected backyard poultry (*39*). An increase in H5N1 virus infections among occupationally exposed poultry workers could signal the emergence of a virus with increased transmissibility among humans (*40*).

Our study has several limitations. First, almost 20% of the poultry workers declined to participate, and 28% of those enrolled at baseline were lost to follow-up. The refusals and losses to follow-up may have led to selection bias, resulting in an underestimation of seroprevalence and incidence of seroconversion if some of these workers were infected or in an overestimation if none of them were infected. Second, once H5N1 virus was detected in surveillance samples from an LBM, we conducted a final follow-up with workers at that LBM. Thus, we may have missed seroconversions that occurred after follow-up. Third, because the modified horse erythrocyte hemagglutination assay is insensitive for the detection of antibody to A/Bangladesh/3233/2011 (H5N1, clade 2.2) virus, we could not use it for confirmation of seropositivity and seroconversion in this study. Fourth, poultry workers in Bangladesh were engaged in multiple activities, making it difficult to identify which specific behavior was the predominant risk factor for H5N1 virus infection. Last, we were unlikely to have accurately ascertained clinical illness associated with H5N1 virus infections because of the lag between collection of H5N1 virus–positive poultry and environmental surveillance samples and the collection of follow-up blood samples from workers.

In conclusion, our study suggests that a low but substantive proportion of LBM poultry workers in Bangladesh become infected with H5N1 virus after unprotected, ongoing sporadic exposures to H5N1 virus–infected poultry and virus-contaminated environments of LBMs. The risk behaviors identified in our study may help public health officials explore interventions to interrupt poultry-to-human transmission of H5N1 virus and other avian influenza A viruses among the poultry workers. The cost of any interventions needs to take into account the anticipated potential modest benefit of decreasing an infrequent event with uncertain pandemic potential.

Technical Appendix 1 Questionnaires administered to the poultry workers and nonpoultry workers during a study of avian influenza A(H5N1) virus infection among workers at live bird markets.

Technical Appendix 2 Workers’ baseline characteristics, exposure to poultry, and use of personal protective equipment; contribution of individual behaviors of poultry workers for each set of exposure behaviors; and timeline for testing.
