# Supplementary material for: Highly Pathogenic Avian Influenza A(H5N1) Virus Infection among Workers at Live Bird Markets, Bangladesh, 2009–2010
Source: Emerg Infect Dis. 2015 Apr;21(4):629–37. doi: 10.3201/eid2104.141281 (PMC4378465; doi:10.3201/eid2104.141281)
Supplement: Technical Appendix 1 — Questionnaires administered to the poultry workers and nonpoultry workers during a study of avian influenza A(H5N1) virus infection among workers at live bird markets. [file 14-1281-Techapp-s1.pdf]

Article DOI: http://dx.doi.org/10.3201/eid2104.141281

Highly Pathogenic Avian Influenza A(H5N1) Virus Infection among Workers at Live Bird Markets, Bangladesh, 2009–2010

Technical Appendix 1

International Centre for Diarrhoeal Disease Research, Bangladesh

Sero-prevalence of antibodies to avian influenza A viruses among poultry market workers

**ID #**						

1. Name of Interviewer:
2. Date: ___ ___ ____ (dd /mm /yy)
3. Location: □ Netrokona □ Chittagong □ Rajshahi □ Dhaka
4. Market ID _________
5. Market worker available (For *follow-up sample collections)*?
   □ Yes □ No
6. Initial visit? □ Yes □ No (if no, skip to 9)
7. Consent to participate? □ Yes (if yes, skip to 9) □ No
8. If refused consent provide reason:______________________ (*Stop and thank interviewee*)

Generic risk factors:

9. How old are you: ____ (years)
10. Sex: □ Female □ Male (if male, skip to 11)
    a. *If female,* Are you pregnant, that you are aware? □ Yes □ No
11. What is your ethnicity:_______
12. Height:______ (meters) *(Use tape measure)*
13. Weight:_____ (Kg) *(Use bathroom scale)*
14. Do you smoke? □ *Yes* □ *No* (if no, skip to 17)
15. How many sticks a day do you smoke____
16. How many years have you smoked? ____
17. Do you use: *(read and select all that apply)*

    □ Betel leaf or betel nuts □ gul (remains of tobacco-cake mixed with molasses)
    □ khoini (hand-made tobacco dust) □ None of the above
18. Has a doctor ever told you that you have any of the following conditions?:

    □ Asthma □ Diabetes □ Chronic heart disease □ Chronic lung disease

□ Chronic kidney disease □ Chronic liver disease □ Immune problems □ Cancer □ None of the above

Environmental risk factors:

19. *Is there any hand washing station in the market (interviewer to observe and record)?* □ *Yes* □ *No*

20. Do you have running water in the market? □ *Yes* □ *No (skip to Q22)*

21. Approximately how far is your water source in the market? ___ (□ *Meters* □ *Feet)(should be blank for the skipped ones)*

22. Did you wash your hands with soap and water while in the market yesterday? □ *Yes* □ *No*

23. Daily, do you use ash or mud to wash your hands? □ *Yes* □ *No*

24. If you washed your hands yesterday, when did you do so: (read all the key times)

Before meals?	□ Always □ Often □ Seldom □ Never
After returning home?	□ Always □ Often □ Seldom □ Never
After defecating?	□ Always □ Often □ Seldom □ Never
Before touching your eyes, nose, or mouth?	□ Always □ Often □ Seldom □ Never

25. Daily, how often do wash your hands with ash or mud: (read all the key times)

Before meals?	□ Always □ Often □ Seldom □ Never
After returning home?	□ Always □ Often □ Seldom □ Never
After defecating?	□ Always □ Often □ Seldom □ Never
Before touching your eyes, nose, or mouth?	□ Always □ Often □ Seldom □ Never

Poultry worker risk factors:

26. Do you handle poultry? □ *Yes* □ *No (if no stop and thank interviewee)*

27. Where do you handle poultry (*check all that apply*)?

□ Home (□ Market □ Farm □ Other _________

28. What kind of tasks do you do when you handle poultry*? (read and select all that apply)*

Transport poultry	□ Daily □ Weekly □ Monthly □ Never
Feed poultry	□ Daily □ Weekly □ Monthly □ Never
Clean feeding tray	□ Daily □ Weekly □ Monthly □ Never
Clean water container	□ Daily □ Weekly □ Monthly □ Never
Slaughter poultry	□ Daily □ Weekly □ Monthly □ Never
Defeather poultry	□ Daily □ Weekly □ Monthly □ Never
Eviscerate poultry	□ Daily □ Weekly □ Monthly □ Never
Collect or transport feces	□ Daily □ Weekly □ Monthly □ Never
Cleaning feces from where poultry are kept	□ Daily □ Weekly □ Monthly □ Never

29. Do you use any personal protective equipment when handling poultry?

Protective apron	□ Always □ Often □ Seldom □ Never
Gloves	□ Always □ Often □ Seldom □ Never

Dedicated coveralls □ Always □ Often □ Seldom □ Never
Mask □ Always □ Often □ Seldom □ Never
Boots □ Always □ Often □ Seldom □ Never

30. Do you eat lunch or drink tea during or after working with poultry? □ Always □ Often □ Seldom □ Never

31. Do you smoke while working with poultry? □ Always □ Often □ Seldom □ Never

32. Do you carry hand poultry or hold poultry on your lap? □ Always □ Often □ Seldom □ Never

33. Do you carry baskets containing poultry on your head? □ Always □ Often □ Seldom □ Never

34. Do you change your clothes upon returning home after working with poultry? □ *Yes* □ *No*

35. Do you eat raw or undercooked poultry or eggs? □ Always □ Often □ Seldom □ Never

Thank you for your cooperation and participation in the survey

Technical Appendix 1 Figure 1. Questionnaire administered to poultry workers at live bird markets, Bangladesh, 2009–2010. The questionnaire was administered to all workers at baseline, and 12 months after baseline to workers at the market where avian influenza A(H5N1) virus was not detected through poultry surveillance during the study.

International Centre for Diarrhoeal Disease Research, Bangladesh

Sero-prevalence of antibodies to avian influenza A viruses among poultry market workers

Market worker questionnaire 21 days after animal surveillance recovers influenza

ID #	1					

1. Name of Interviewer:
2. Date: ___ ___ ____ (dd mm yy)
3. Location: □ Netrokona □ Chittagong □ Rajshahi □ Dhaka
4. Market ID _________
5. Market worker available? □ Yes □ No

Influenza like illness:

6. Have you been sick in the past 21 days? □ Yes □ No **(if no, skip to 22)**
7. When did you first feel sick? Date __ __ __ (dd mm yy)
8. Did you develop a sudden fever? □ Yes □ No
9. Did someone take your temperature? □ *Yes* □ No **(if no, skip to 11)**
10. What was your highest temperature? ____ F^0
11. Did you develop:
    a. Cough? □ Yes □ No
    b. Sore throat? □ Yes □ No
    c. Shortness of breath or difficulty breathing? □ Yes □ No
12. Did you seek medical attention? □ Yes □ No **(if no, skip to 19)**
13. Where did you seek medical attention? □ Local clinic □ Local hospital □ Other__________
14. What were you diagnosed with?
    d. □ Cold □ Pharyngitis □ Bronchitis □ Pneumonia □ Dengue □ Other___________
15. Were you told you needed hospitalization? □ Yes □ No
16. Have you taken oseltamavir (show case-patient sample blister pack) for this illness as twice a day for 5 days (*or up to the time of the interview*)? □ Yes □ No
17. Did a doctor obtain a clinical sample?
    e. From nose or throat □ Yes □ No
    f. Blood □ Yes □ No } **(if no, skip to 19)**
18. Where was this sample obtained?:_______________________
19. In the 3 days before symptom onset, had anyone **at home** had similar symptoms? □ Yes □ No
    g. If yes, who__________________

20. In the 3 days before symptom onset, did you know of anyone with similar symptoms?
□ *Yes* □ *No* **(if no Skip to 22)**

21. In the 3 days before symptom onset, had you been close (< 3 feet/ 2 hands) to anyone you know with similar symptoms **outside** the home? □ *Yes* □ *No* **(if no Skip to 22)**

    h. If yes, where (check all that apply):
    □ Market □ School □ Mosque/church/temple □ Street □ Other home
    □ Other______________________

Potential risk factors for present illness

22. In the 3 days before symptom onset/7 days before collecting the animal sample (mention the date), had you been around sick poultry? □ *Yes* □ *No*

23. Did you handle the sick poultry? □ Yes □ No **(if no skip to 34)**

24. Where did you handle sick poultry (*check all that apply)*?
□ Home (H) □ Market (M) □ Farm (F) □ Other _________

25. What kind of tasks did you do when you handle the **sick poultry** and where *(check all that apply and add location code [i.e. H,M,F])?*

Transport poultry	□ *Yes*	□ *No*	Location:_______
Feed poultry	□ *Yes*	□ *No*	Location:_______
Clean feeding tray	□ *Yes*	□ *No*	Location:_______
Clean water container	□ *Yes*	□ *No*	Location:_______
Give medicine to the sick poultry	□ *Yes*	□ *No*	Location:_______
Separate sick poultry	□ *Yes*	□ *No*	Location:_______
Slaughter poultry	□ *Yes*	□ *No*	Location:_______
Defeather poultry	□ *Yes*	□ *No*	Location:_______
Eviscerate poultry	□ *Yes*	□ *No*	Location:_______
Collect or transport feces	□ *Yes*	□ *No*	Location:_______
Cull poultry	□ *Yes*	□ *No*	Location:_______
Stuff poultry in bags	□ *Yes*	□ *No*	Location:_______
Bury poultry carcasses	□ *Yes*	□ *No*	Location:_______
Burn poultry products	□ *Yes*	□ *No*	Location:_______
Cleaning feces from place where poultry are kept	□ *Yes*	□ *No*	Location:_______

26. Did you take precautions when handling ill poultry (check all that apply)?

Protective apron	□ Always □ Often □ Seldom □ Never
Gloves	□ Always □ Often □ Seldom □ Never
Dedicated coveralls	□ Always □ Often □ Seldom □ Never
Mask	□ Always □ Often □ Seldom □ Never
Boots	□ Always □ Often □ Seldom □ Never

27. Did you eat during or after working with ill poultry? □ *Yes* □ *No*

28. Did you smoke while working with ill poultry? □ *Yes* □ *No*

29. Did you use: *(read and select all that apply)*

□ Betel leaf or betel nuts
□ gul (remains of tobacco-cake mixed with molasses)
□ khoini (hand-made tobacco dust)
□ None of the above

30. Did you hand carry sick poultry or hold poultry on your lap? □ *Yes* □ *No*

31. Did you carry baskets containing sick poultry on your head? □ *Yes* □ *No*

32. Did you wash your hands at the market after working with ill poultry? □ *Yes* □ *No*

33. Did you change your clothes upon returning home after working with ill poultry? □ *Yes* □ *No*

34. Did you eat raw or undercooked poultry or eggs? □ *Yes* □ *No*

Thank you

Technical Appendix 1 Figure 2. Questionnaire administered to poultry workers at follow-up during a study of avian influenza A(H5N1) virus infection among workers at live bird markets, Bangladesh, 2009–2010.

International Centre for Diarrhoeal Disease Research, Bangladesh

Sero-prevalence of antibodies to avian influenza A viruses among poultry market workers

Non-poultry workers questionnaire

ID #						

1. Name of Interviewer:
2. Date: ___/ ___/ ____ (dd/mm/yy)
3. Name of the Organization of control:
4. Consent to participate? □ Yes (if yes, skip to 7) □ No
5. If refused consent provide reason:_______________________ (*Stop and thank interviewee*)

Generic risk factors:

6. How old are you: ___ ___ (years)
7. Have you owned or handled poultry during the past 2 years? □ *Yes (if yes thank and stop)* □ *No*
8. Have you worked in influenza field studies during past 2 years? □ *Yes (if yes thank and stop)* □ *No*
9. Sex: □ Female □ Male (if male, skip to 11)
   a. *If female,* Are you pregnant, that you are aware? □ Yes □ No
10. What is your ethnicity:_______
11. Height:______ (meters) *(Use tape measure)*
12. Weight:_____ (Kg) *(Use bathroom scale)*
13. Do you smoke? □ *Yes* □ *No* (if no, skip to 18)
14. How many sticks a day do you smoke____
15. How many years have you smoked? ____
16. Do you use: *(read and select all that apply)*

    □ Betel leaf or betel nuts □ Gul (remains of tobacco-cake mixed with molasses)
    □ Khoini (hand-made tobacco dust) □ None of the above

17. Do you have any of the following conditions?:

    □ Asthma □ Diabetes □ Chronic heart disease □ Chronic lung disease

□ Chronic kidney disease □ Chronic liver disease □ Immune problems □ Cancer □ None of the above

Environmental risk factors:

18. Daily, do you use soap to wash your hands? □ *Yes* □ *No*

19. Daily, do you use ash or mud to wash your hands? □ *Yes* □ *No*

20. Daily, how often do wash your hands: (read all the key times)

Before meals?	□ Always □ Often □ Seldom □ Never
After returning home?	□ Always □ Often □ Seldom □ Never
After defecating?	□ Always □ Often □ Seldom □ Never
Before touching your eyes, nose, or mouth?	□ Always □ Often □ Seldom □ Never

Poultry risk factors:

21. Have you ever handled poultry? □ *Yes* □ *No* (*Stop and thank interviewee*)

22. Where have you handled poultry (*check all that apply*)?

□ Home □ Market □ Farm □ Other _________

Thank you

Technical Appendix 1 Figure 3. Questionnaire administered to nonpoultry workers during a study of avian influenza A(H5N1) virus infection among workers at live bird markets, Bangladesh, 2009–2010.
