# Supplementary material for: Highly Pathogenic Avian Influenza A(H5N1) Virus Infection among Workers at Live Bird Markets, Bangladesh, 2009–2010
Source: Emerg Infect Dis. 2015 Apr;21(4):629–37. doi: 10.3201/eid2104.141281 (PMC4378465; doi:10.3201/eid2104.141281)
Supplement: Technical Appendix 2 — Workers’ baseline characteristics, exposure to poultry, and use of personal protective equipment; contribution of individual behaviors of poultry workers for each set of exposure behaviors; and timeline for testing. [file 14-1281-Techapp-s2.pdf]

Article DOI: http://dx.doi.org/10.3201/eid2104.141281

Highly Pathogenic Avian Influenza A(H5N1) Virus Infection among Workers at Live Bird Markets, Bangladesh, 2009–2010

Technical Appendix 2

Technical Appendix 2, Table 1. Baseline characteristics among poultry workers, by follow-up status, in a study of avian influenza A(H5N1) virus infection among workers at live bird markets, Bangladesh, 2009–2010*

	No. (%) poultry workers		
Baseline characteristic	Followed up, n = 290	Lost to follow-up, n = 114	p value
Median age, y (IQR)	27 (22–38)	28 (23–35)	0.9†
Smoker	166 (57)	70 (61)	0.4‡
Have chronic medical condition§	18 (6)	10 (9)	0.4‡
Exposure to poultry			
Transport poultry	204 (70)	86 (75)	0.3‡
Feed poultry	226 (78)	72 (63)	0.002‡
Clean feeding tray	177 (61)	66 (58)	0.6‡
Clean water container	168 (58)	63 (55)	0.6‡
Slaughter poultry	229 (79)	75 (66)	0.006‡
Defeather poultry	180 (62)	66 (58)	0.4‡
Eviscerate poultry	178 (61)	65 (57)	0.4‡
Collect or transport feces	61 (21)	21 (18)	0.6‡
Clean feces from pens	137 (47)	51 (45)	0.7‡
Hand-carry poultry or hold poultry on lap during travel	265 (91)	111 (97)	0.03‡
Carry baskets containing poultry on head	4 (1)	8 (7)	0.003‡
Eat raw or undercooked poultry or eggs	84 (29)	47 (41)	0.02‡
Use of personal protective equipment¶			
Protective apron	0 (0)	0 (0)	–
Gloves	0 (0)	0 (0)	–
Dedicated overalls	0 (0)	0 (0)	–
Cloth mask	5 (2)	1 (1)	0.5
Boots	1 (0.37)	0 (0)	0.5

*IRQ, interquartile range; –, not applicable.
†p value for 2-sample Wilcoxon rank-sum test.
‡p value for 2-sample test of proportion.
§Conditions such as asthma; diabetes; chronic heart, lung, kidney, liver, and kidney disease; immune disorder; and cancer.
¶During reported handling of poultry at baseline or handling of sick poultry at follow-up for the followed up workers; and handling of poultry at baseline for the lost to follow-up workers.

Technical Appendix 2, Table 2. Contribution of individual behaviors of poultry workers for each set of exposure behaviors (factor loadings) associated with avian influenza A(H5N1) virus infection among live bird market workers, Bangladesh 2009–2010*

	Behavior		
Exposure behaviors	Medium exposure	High exposure	Low exposure
Smoked	–0.0097	–0.0018	0.1193
Fed poultry	0.3628	0.5927	0.0646
Cleaned feeding tray	0.3670	0.8982	0.0150
Cleaned water container	0.3659	0.8958	0.0628
Medicated poultry	0.0693	0.1218	0.2189
Isolated sick poultry	0.1925	0.2104	0.4459
Slaughtered poultry	0.5669	0.3162	0.1107
Defeathered poultry	0.9270	0.3308	0.0413
Eviscerated poultry	0.9179	0.3550	0.0380
Collected or transported feces	0.2626	0.2538	0.1016
Stuffed poultry into bags	0.6055	0.2928	0. 0581
Cleaned feces from pens	0.4939	0.4968	–0.0036
Did not wash hands after handling sick poultry	–0.1326	–0.0432	–0.4368
Ate raw or undercooked poultry or eggs	0.0280	0.0766	0.1892

*H5N1 virus infection denotes being seropositive at baseline/follow-up and having evidence of seroconversion to H5N1 virus antibodies at follow-up. Gray shedding shows that exposure behaviors with the highest factor loading were grouped together into each set of exposure behaviors that were later classified into high-, medium- and low-exposure on the basis of calculated risk ratios.

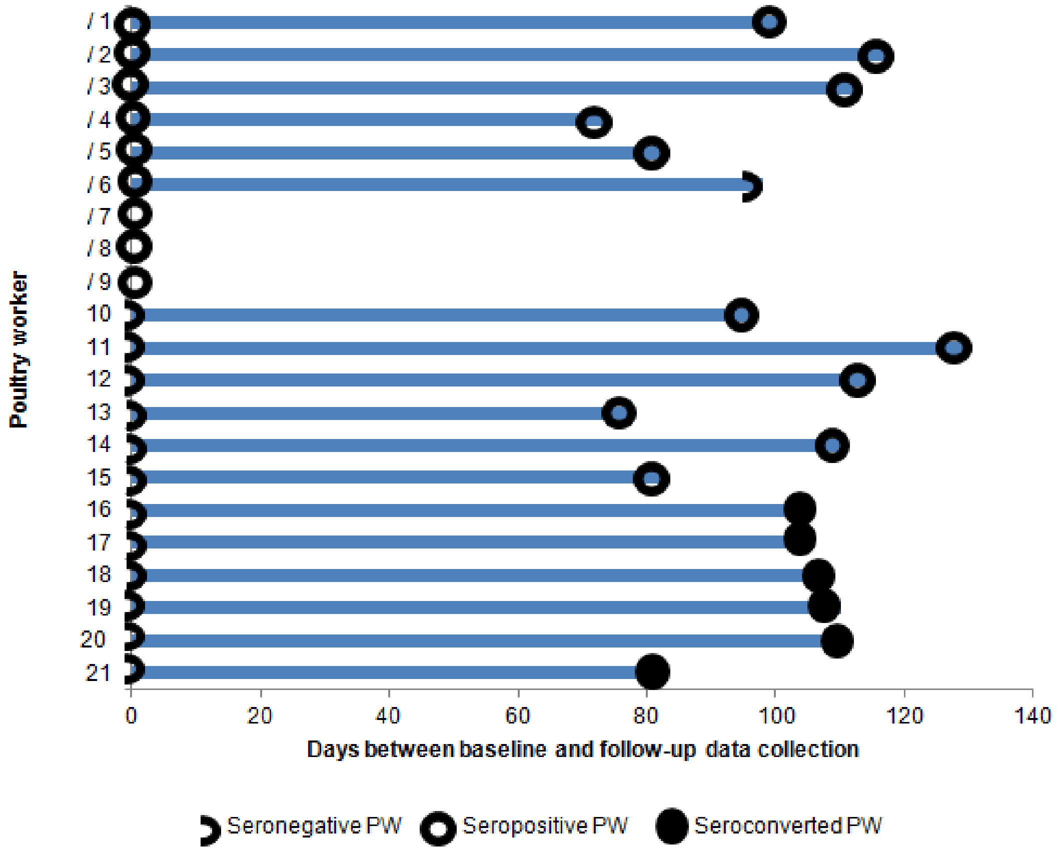

Technical Appendix 2, Figure. Timeline for the collection of serum samples for 21 poultry workers (PWs) who were positive for influenza A(H5N1) virus at baseline or who seroconverted at follow-up. The samples were obtained for A(H5N1) virus serologic testing.
